# Elevated Serum Levels of Retinol-Binding Protein 4 Are Associated with Breast Cancer Risk: A Case-Control Study

**DOI:** 10.1371/journal.pone.0167498

**Published:** 2016-12-21

**Authors:** Congcong Jiao, Lianhua Cui, Aiguo Ma, Na Li, Hongzong Si

**Affiliations:** Department of Public Health, Medical College of Qingdao University, Qingdao, Shandong Province, China; University of South Alabama Mitchell Cancer Institute, UNITED STATES

## Abstract

**Background:**

Retinol binding protein 4 (RBP4) is a recently identified adipokine that is elevated in patients with obesity or type 2 diabetes. A growing body of research has shown that RBP4 is associated with several types of cancer. However, no studies have investigated the relationship between serum RBP4 levels and breast cancer risk. We performed a case-control study to evaluate the association between serum RBP4 levels and the risk of breast cancer.

**Methods:**

From August 2012 to December 2013, four-hundred subjects including 200 patients diagnosed with primary breast cancer and 200 matched healthy women were consecutively enrolled from Affiliated Hospital of Qingdao University Medical College. Blood samples were collected from healthy controls and breast cancer patients before commencement of treatment. Enzyme-linked immunosorbent assay was used to evaluate the serum RBP4 levels in separated serum samples. Meanwhile, the characteristics of breast cancer cases and controls were collected from medical records and pathological data.

**Results:**

The serum levels of RBP4 were significantly higher in patients with breast cancer than that in the healthy control group (33.77±9.92 vs. 28.77±6.47μg/ml, P < 0.05). Compared to the subjects in the lowest quartile of serum RBP4 level, the adjusted ORs (95% CIs) is 2.16(1.01–4.61) and 2.07 (1.07–4.00) for women in the second and highest RBP4 tertile, respectively. For breast cancer patients, patients with PR or ER negative displayed significantly higher serum RBP4 levels than those with PR or ER positive.

**Conclusion:**

Our results for the first time suggested serum RBP4 levels could be associated with the risk of breast cancer. However, further prospective studies are essential to confirm these observed results.

## Introduction

Breast cancer is the most common malignancy tumor and is the main cause of cancer deaths among women worldwide, with an estimated 1.67 million new cases in 2012 (25 percent of the total cases of cancers)[1]. The incidence and mortality of breast cancer among Chinese women has steadily increased in the past years[2]. In fact, in the last 25 years, the incidence of breast cancer has risen by more than 51% in major Chinese cities including Beijing, Shanghai, and Guangzhou[3].

The occurrence and development of breast cancer is a complicated dynamic process with many reported risk factors, and one of the established risk factors for breast cancer developments is obesity[4, 5]. Adipose tissue is not only an energy reservoir pool, but also a major endocrine function releasing multiple adipokines including leptin, adiponectin, tumor necrosis factor-α, interleukin-6 and resistin[6], these adipokines contribute to the pathogenesis of obesity-linked complications such as insulin resistance, type 2 diabetes, metabolic syndrome and breast cancer[7–10]. Retinol binding-protein 4 (RBP4) is a recently identified adipokine, many studies have declared the relationship between the increased circulating RBP4 and different aspects of obesity[11–14]. However, no studies have investigated the relationship between RBP4 and breast cancer risk.

RBP4, a novel adipokine, belongs to the lipocalin family of proteins that transport small hydrophobic molecules. RBP4 is a 21-KDa protein and the protein encoder gene is located on chromosome 10 (10q23.33)[15]. Previously, RBP4 was known as the specific transport protein that transports retinol (vitamin A) from the liver to the peripheral tissue. In peripheral tissues, RBP4 may act directly by binding to cell surface receptors[16]or through retinoic acid on retinoic acid receptors and retinoic acid-X receptors[17]. Liver has the highest expression level of RBP4 and adipose tissue has the second highest rate of expression. In adipose tissue, RBP4 is expressed in mature, lipid-laden adipocytes[18]. Subsequently, it has been recognized a novel adipokine since a study on adipose-specific glucose transporter type 4 (Glut4)knockout mice in 2005[19]. Just as other adipokines, a growing body of research has shown that elevated RBP4 levels were positively correlated with obesity-linked complications including impaired glucose tolerance, insulin resistance, type 2 diabetes mellitus, dyslipidemia, hypertension and cardiovascular diseases[20–22]. Regarding the relationship between serum RBP4 levels and cancer, Uehara et al.[23] reported that RBP4 was associated with prostate cancer cell growth in vitro and increased in prostate cancer cells. In addition, epidemiological study showed that higher circulating levels of RBP4 are associated with colon adenoma[24], ovarian cancer[25] and oral squamous cell cancer[26]. Moreover, Wang et al. [27] also found that RBP4 protein is correlated with overall survival of patients with hepatocellular carcinoma.

To the best of our knowledge, no studies have investigated the relationship between RBP4 and the risk of breast cancer. Hence, the purpose of the present study was to determine whether high levels of serum RBP4 are associated with an increased risk of breast cancer using a case–control study. In addition, we also investigated the association between serum RBP4 levels and clinicopathological features of breast cancer.

## Materials and Methods

### Study subjects

From August 2012 to December 2013, a case-control study was conducted at Affiliated Hospital of Qingdao University Medical College. Four-hundred patients including 200 cases and 200 matched controls were consecutively enrolled in our study. Each subject signed standard consent statement before being recruited into this study. The study protocol was approved by Qingdao university medical college ethics committee. Cases were women with a diagnosis of primary breast cancer recruited consecutively from individuals visiting breast surgeon of Affiliated Hospital of Qingdao University Medical College. The inclusion criteria for cases were as follows: 1) diagnosing with primary breast cancer within six months, 2) confirmed by histopathology, 3) no receiving chemotherapy, radiotherapy, surgery, or blood transfusions, 4) having complete pathological data and medical records. Diagnosed patients who suffered from serious complications or other types of cancer were not included. Then, the pathology report from each case was reviewed to confirm the diagnosis. In the same period, 200 female subjects (controls) without any clinical signs, symptoms or suspicion of any type of cancer in their medical history were was randomly selected from physical examination center of Affiliated Hospital of Qingdao University Medical College. Efforts were made to frequency match the cases and controls by age (within 5-year intervals).

Subjects’ characteristics including age, BMI, age at menarche, menopausal status and history of abortion were collected from medical records. Tumor size, pathological type, metastasis status and the status of p53, estrogen receptor (ER) and progesterone receptor (PR) of 200 breast cancer specimens were obtained from pathological data. The pathological tumor stage was defined according to the 7th edition of the tumor-node-metastasis (TNM) classification of the American Joint Committee on Cancer (AJCC).

### Biochemical measurements

Blood samples of patients were drawn before initiating chemo (radio)therapy and/or surgical resection of tumor. After at least 12 hours fasting, blood samples were collected from breast cancer patients and healthy controls. To separate serum from blood cells, samples were centrifuged at 3,000g for 10 min at 4°C following 30-min clotting. Freshly separated serum was employed for determination of lipid profile and blood glucose, and the remaining aliquots of serum were stored at −80°C until they were assessed for serum RBP4 levels. The assay of triglycerides (TC), high-density lipoprotein (HDL), low-density lipoprotein (LDL), triglycerides (TG) and fasting blood glucose (FBG) were carried out in an automated analyzer (Cobas Integra 400, Roche Diagnosis). Serum RBP4 was estimated by an enzyme-linked immunosorbent assay (ELISA) (R&D Systems China Co. Ltd) with the lower detection limit of 0.048 mM/l. The inter- and intra-assay coefficients of variation were 7.2% and 6.9% respectively.

### Statistical analysis

All the data were statistically analyzed using the SPSS 17.0 software and the significance level was set at p ≤ 0.05. Data are presented as means±SD as the data was normally distributed. To compare variables between two groups or subgroups, Chi-square tests for categorical variables and Student’s t-test or one-way ANOVA for continuous variables were used. Multivariate logistic regression models were used to assess the risk of breast cancer according to the tertiles of serum RBP4 level. Multivariate linear regression analysis were used to determine the relationships between BMI, TC, TG, HDL, LDL, FBG and serum RBP4.

## Results

Characteristics of cancer patients and healthy controls are summarized in Table 1. As subjects in two groups have been matched by age, no significant differences were observed for age between healthy controls and breast cancer patients. Compared to controls, more subjects of case group have higher menarche age and BMI (P <0.05), while there was no statistically significant difference on menopausal status and history of abortion. As regards to blood lipids indices, the serum level of TG, TC, and LDL in breast cancer patients have lower level of HDL(P <0.05). No significant differences were observed between the 2 groups regarding the serum level of FBG. The mean RBP4 levels (mean ±SD) of patients and controls were 33.77±9.92μg/ml and 28.78±6.43μg/ml, respectively. The serum RBP4 levels in case group was significantly higher than that in control group (P < 0.05).

**Table 1:** Characteristics of breast cancer cases and controls.

Variables	Case(n = 200) n(%)	Control(n = 200) n(%)	P value
**Age(years)**			
<55	143(71.5%)	112(56%)	0.072
≥55	57(28.5%)	88(44.0%)	
**Miscarriage**			
Yes	24(12.0%)	19(9.5%)	0.420
No	176(88.0%)	181(90.5%)	
**Menarche Age(years)**			
<14	38(20.1%)	48(24.1%)	0.001
14–15	67(35.4%)	98(49.2%)	
≥16	84(44.4%)	53(26.6%)	
Unknown	11(5.5%)	1(0.5%)	
**Menopause**			
Yes	129(64.5%)	131(65.5%)	0.676
No	71(35.5%)	69(34.5%)	
**BMI(kg/m**^**2**^**, Mean±SD)**	24.68±3.13	23.84±3.23	0.008
**Serum Markers(Mean±SD)**			
TC(mmol/L)	5.06±0.93	4.56±1.03	0.000
TG(mmol/L)	1.65±1.10	1.33±0.81	0.001
HDL(mmol/L)	1.36±0.31	1.95±0.57	0.000
LDL(mmol/L)	2.75±0.69	2.42±0.71	0.000
FBG(mmol/L)	5.31±1.57	5.13±1.58	0.258
RBP4(ug/ml)	33.77±9.92	28.77±6.47	0.000

SD, standard deviation; BMI, body mass index; TG, triglycerides; TC, total cholesterol; HDL, high-density lipoprotein; LDL, low-density lipoprotein; FBG, fasting blood glucose; RBP4, Retinol-Binding Protein 4.

Table 2 exhibits the results of the multivariate unconditional logistic regression analysis. We compute the odds ratios (ORs) and 95% confidence intervals (CIs) for breast cancer by tertile of RBP4 levels, which is defined by the serum RBP4 levels of healthy subjects. After adjustment for age and menarche age, the OR of breast cancer is 2.44 in individuals with the highest RBP4 tertile, when compared with individuals with the lowest RBP4 tertile(*P* <0.05). Subjects in the second RBP4 tertile also revealed increased risk of breast cancer and the OR is 2.03(*P* <0.05). When the model was further adjusted for potential risk factors of breast cancer, the results were similar, with ORs (95% CIs) of 2.16(1.01–4.61) and 2.07(1.07–4.00) for women in the second and highest RBP4 tertile, respectively, when compared to those in the lowest tertile(*P* <0.05).

**Table 2:** Logistic Regression Analysis of Risk of Breast cancer for Serum Levels of RBP4.

RBP4^a^(ug/ml)	Cases n (%)	Control n(%)	OR ^b^ (95% CI)	*P*	OR^c^(95% CI)	*P*
T1 (<24.4)	38(19.0%)	68(34.0%)	1(Reference)		1(Reference)	
T2 (24.4–31.3)	47(23.5%)	41(20.5%)	2.03(1.11–3.69)	0.021	2.16(1.01–4.61)	0.048
T3(≥31.4)	115(57.5%)	91(45.5%)	2.44(1.47–4.05)	0.001	2.07(1.07–4.00)	0.030

RBP4, Retinol-Binding Protein 4; OR, odds ratio

a: Tertile distribution based on the tertile distribution of RBP4 level in control group

b: Adjusted for age and menarche age

c: Adjusted for age, menarche age, triglycerides, cholesterol, body mass index, high-density lipoprotein, low-density lipoprotein.

Table 3 shows partial correlation coefficient (β) for RBP4 and metabolism indexes in the breast cancer group and healthy group. After adjusted for age,miscarriage history, menopause status and menarche age, there was significantly positive correlation between serum RBP4 level and BMI in breast cancer group(*P* < 0.05), while circulating RBP4 levels were significantly correlate with BMI and TG in control group. Both in two groups, we fail to found significant relationship between RBP4 and TC, HDL, LDL or FBG.

**Table 3:** Partial correlation coefficient (β) for RBP4 and metabolism indexes.

Indexes	Case(n = 200)		Control(n = 200)	
	β^a^-Value	p-Value	β^a^-Value	p-Value
**BMI**	0.214	0.004	0.145	0.043
**HDL**	0.034	0.181	-0.093	0.198
**LDL**	0.031	0.674	0.010	0.888
**TC**	0.061	0.413	-0.004	0.951
**TG**	0.079	0.285	0.149	0.037
**FBG**	0.000	0.992	0.012	0.867

BMI, body mass index; TG, triglycerides; TC, total cholesterol; HDL, high-density lipoprotein; LDL, low-density lipoprotein; FBG, fasting blood glucose

a: Adjusted for age, miscarriage history, menopause status and menarche age.

To further evaluate the association of likelihood of breast cancer risk with serum RBP4 levels, we performed a stratified analysis by BMI (Table 4).In the subgroup of higher BMI(≥25kg/m^2^), serum RBP4 levels was not significantly associated with the risk of breast cancer. Whereas, serum RBP4 levels were positively associated with breast cancer risk among subjects with lower BMI (<25 kg/m^2^) (multivariable OR = 2.72, 95% CI = 1.09 to 6.79 for second versus lowest tertile).

**Table 4:** Stratified analyses of Odds ratios and 95% confidence intervals of breast cancer with RBP4.

BMI (kg/m^2^)	RBP4(ug/ml)	Cases n (%)	Control n(%)	OR^a^ (95%CI)	*P*
**<25**	T1	23(20.2%)	48(37.5%)	1(Reference)	
	T2	31(27.2%)	22(17.2%)	2.72(1.09–6.79)	0.033
	T3	60(52.6%)	58(45.3%)	2.08(0.95–4.53)	0.066
**≥25**	T1	15(17.4%)	20(27.8%)	1(Reference)	
	T2	16(18.6%)	19(26.4%)	1.45(0.34–6.25)	0.620
	T3	55(64.0%)	33(45.8%)	2.45(0.66–9.16)	0.183

BMI, body mass index

OR^**a**^, odds ratio adjusted for age, menarche age, triglycerides, cholesterol, high-density lipoprotein, low-density lipoprotein.

Table 5 shows the relationship between serum RBP4 levels and clinicopathological features in case group. Patients were divided into subgroups, according to presence or absence of metastasis, histological grade of cancer, histopathological subtypes, positive and/or negative results for ER, PR and p53. We analyzed the mean of serum RBP4 levels in the classified breast cancer patients. Compared to patients with PR or ER positive, patients with PR or ER negative displayed significantly higher serum RBP4 levels. There was no significant difference on the mean serum RBP4 levels of the breast cancer in p53 status, histopathological subtype, clinical stage and metastasis status.

**Table 5:** Correlation between Serum RBP4 and clinical characteristics in breast cancer patients.

Characteristics	Number (%)	RBP4 (Mean±SD, ug/ml)	*P* value
**ER**			0.041
Negative	106(53.0%)	35.12±10.31	
Positive	94(47.0%)	32.24±9.28	
**PR**			0.027
Negative	104(52.0%)	35.25±10.41	
Positive	96(48.0%)	32.16±9.15	
**P53**			
Negative	96(48.0%)	32.67±9.85	0.134
Positive	104(52.0%)	34.78±9.92	
**Histopathological subtype**			0.584
IDC	145(72.5%)	33.83±9.37	
ILC	33(16.5%)	32.24±10.36	
Others	19(9.5%)	35.15±9.61	
**TNM stage**			0.117
Ⅰ+Ⅱ	124(62.0%)	32.91±10.09	
Ⅲ+Ⅳ	76(38.0%)	35.17±9.54	
**Metastasis**			0.733
No	146(73.0%)	33.91±9.98	
Yes	54(27.0%)	33.37±9.83	

SD, standard deviation; RBP4, Retinol-Binding Protein 4; IDC, invasive ductal carcinoma; ILC, invasive lobular carcinoma; ER, estrogen receptor; PR, progesterone receptor.

## Discussion

In this current case-control study, we observed a positive correlation between the levels of serum RBP4 and breast cancer risk, especially among patients with lower BMI. These associations were largely independent of established breast cancer risk factors, including age, menarche age and BMI and blood-lipoid indexes. Among breast cancer patients, serum RBP4 levels were significant higher in patients with ER or PR negative than those in patients with ER or PR positive.

Most of the adipokines have been linked to the risk of various kinds of cancer such as gastric malignancy[28], hepatocellular carcinoma[29], prostate cancer[30] and breast cancer[31, 32]. RBP4 as a novel adipokine, it has also been associated with obesity-related co-morbidities including insulin resistance, dyslipidemia, type 2 diabetes and coronary heart disease [19, 20, 33, 34], while these obesity-related diseases are well-established risk factor for breast cancer. However, the evidences on associations of serum RBP4 with breast cancer risk have not been well studied.

Several studies have showed that RBP4 has a role in some types of cancers such as lung[35], pancreatic[36], ovarian[25], squamous cell[26], colorectal[24], prostate cancer[23] and hepatocellular carcinoma[27]. For example, Hisanori Uehara et al[23] showed that RBP4 expression is increased in prostate cancer cells and associated with prostate cancer cell growth in vitro study. Wang et al. observed the over expression of RBP4 protein in HCC cell lines compared to normal livers (P < 0.001), which correlated with metastatic potential[27]. In addition, Cheng et al[25] indicated RBP4 expression levels in normal ovarian tissue and plasma of healthy individuals were lower than those in ovarian cancer tissues and plasma of ovarian cancer patients. Moreover, Patz et al [35] and Chen et al[26] suggested RBP4 might be used as a biomarker for the diagnosis of lung cancer and oral squamous cell carcinoma. Higher circulating RBP4 levels are associated, independent of insulin resistance and diabetes mellitus, with the risk of colon adenoma[24]. Similarly, our results showed a strong positive association between circulating RBP4 levels and breast cancer risk. This association was independent of BMI, lipids, and glucose, suggesting that mechanisms other than components of metabolic syndrome may contribute to the link between RBP4 and breast cancer. A recent study has shown that the holo-RBP protein may act through its own receptor STRA6, which transduces JAK2/STAT3/5 signaling, to initiate carcinogenic transformations[37].

Previous studies revealed a correlation between serum RBP4 levels and obesity, dyslipidemia and FPG. Two studies in Chinese population have reported that RBP4 levels are positively associated with BMI, TG, TC, LDL and FBG while negatively related to HDL[38, 39]. However, some studies found that serum RBP4 levels were not relevant with all of these parameters of metabolic syndrome. One study revealed a significant positive correlation between serum RBP4 levels and FPG, no significant relationship was observed between RBP4 and other factors[22]. Another study showed that serum RBP4 levels were correlated with elevated TG and reduced HDL levels, but not with TC, LDL, FBG and BMI [40]. Our results suggested that serum RBP4 levels are positively correlated with BMI and TG among healthy control group, serum RBP4 concentrations were positively correlated with BMI among patients with breast cancer.

Obesity have been closely related to occurrence and development of breast cancer[41, 42], while elevated RBP4 levels were directly correlated with obesity[43]. Thus, we further conducted a stratified analysis by BMI, and the results indicated that breast cancer risk have a positive association with second tertile of serum RBP4 level in lower BMI subgroup(BMI <25kg/m^2^). In the higher BMI (≥25kg/m^2^) subgroup, there is no significant association of serum RBP4 with the risk of breast cancer. This result is agreement with the previous study on RBP4 and risk of colon adenoma [24], they also found that there is significant association of serum RBP4 and the risk of colon adenoma in lower BMI group, but not in higher BMI group. These results further indicated that RBP4 is associated with cancer risk independent of BMI.

Several epidemiological studies have reported that serum lipid abnormalities, including elevated total cholesterol, LDL-cholesterol and triglycerides and reduced HDL-cholesterol, increase the risk of breast cancer[44–46]. In the present study, we also found higher BMI, elevated TC, TG and LDL levels and reduced HDL levels in patients with breast cancer than those in healthy controls, which is consistent with previous study [44–46]. Moreover, it also has been reported that higher levels of fasting plasma glucose have been associated with an increased breast cancer risk [47, 48]. However, some studies presented a different perspective that no significant association was found between serum glucose levels and breast cancer[49, 50]. A meta-analysis conducted by Boyle et al[51] also revealed that the risk of breast cancer associated with fasting serum glucose levels seems to be small. In the present study, we found no significant differences between patients with breast cancer and healthy control groups in FBG levels.

In this study, we also analyzed the association between serum RBP4 levels and clinical characteristics among breast cancer patients. The results showed that the high levels of serum RBP4 is associated with negative ER and PR status, but not with histopathological subtype, TNM stage, metastasis and P53 status. ER and PR are hormone receptors on breast cell that receive hormone signals informing the cell to grow. The expression status of these hormone receptors in breast cancer is associated with clinical and biological heterogeneity[52]. Many studies have suggested that ER/PR-positive breast cancers are well differentiated and have a better outcome compared to other subtypes[53, 54]. Although data regarding the relationship between RBP4 and estrogen are unavailable, there is evidence that estrogen signaling pathways are interrelated with insulin and other adipokines including leptin, tumor necrosis factor-a and interleukin-6[31, 55]. M et al[56] revealed a significant relationship between the ER/PR status and the serum leptin levels. Adel et al.[57] also observed that three adipokines including leptin, resistin and visfatin levels were significantly higher in negative ER or PR cases. Similarly, Milikan et al[58] and Mahle et al[59] found a positive association between BMI and hormone receptor-negative tumors. Whereas Yang et al[60] found that BMI seems to be more associated with hormone receptor positive tumors rather than triple negative breast cancer (TNBC). Our result revealed that the serum level of RBP4 was significantly higher in patients with ER or PR negative than those with ER or PR positive, which indicated that high serum RBP4 levels could be a risk factor for poor prognosis of breast cancer. Previous study conducted by wang et al. [27] also found that RBP4 protein is correlated with metastatic potential and overall survival of patients with hepatocellular carcinoma.

Dietary pattern have been closely related to the risk of breast cancer[61], while dietary intake of many nutrients can affect serum RBP4 concentration[62]. Due to a lack of detailed data on dietary information, the possible influence of diet on the association of serum RBP4 with breast cancer could not be adequately evaluated. Furthermore, as the limitation of case-control study, it may be difficult to establish the timeline of exposure to disease outcome in the setting of a case-control study. In other words, it is hard to define the causal relationship between serum RBP4 levels with breast cancer. Therefore, in the future prospective cohort studies on serum RBP4 and breast cancer should be designed to further ascertain this relationship.

In summary, the results from this study show that higher circulating levels of RBP4 are associated, independent of BMI, lipids, FBG and other potential risk factors, with an increased risk of breast cancer. We infer that serum RBP4 level may be correlated with breast cancer prognosis. Further investigation is merited to corroborate our findings and to elucidate the underlying molecular mechanism between RBP4 and breast cancer.

## Supporting Information

S1 Table Relevant data underlying the findingsdescribed in manuscript. (XLSX)
